# Supplementary material for: Bayesian inference of protein structure from chemical shift data
Source: PeerJ. 2015 Mar 24;3:e861. doi: 10.7717/peerj.861 (PMC4375973; doi:10.7717/peerj.861)
Supplement: Supplemental Information 2 [file peerj-03-861-s002.pdf]

SUPPLEMENTARY INFORMATION

Lowest energy structures

Below is shown the cluster representative structures referred to in the main text, visualized with PyMOL (Schrödinger, LLC, 2010).

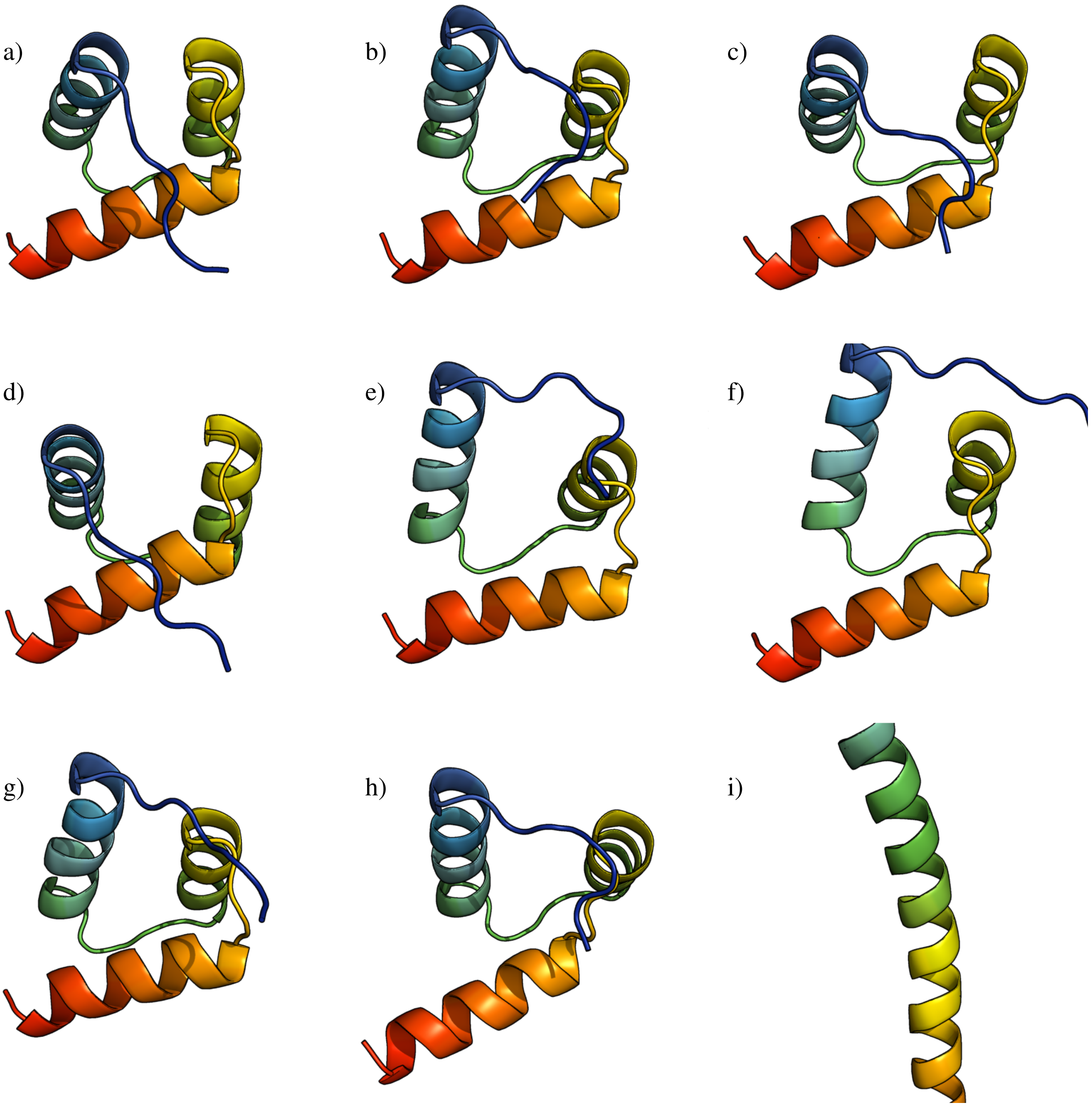

Figure S1. Lowest PROFASI+CamShift energy structures for ENHD. a) Crystal structure 1ENH. b) Cauchy / fixed. c) Cauchy / sampled. d) Gaussian / sampled. e) Gaussian / fixed. f) Gaussian / marginalized. g) Square well / $\alpha = 1$. h) Square well / $\alpha = 5$. i) CS-Torus

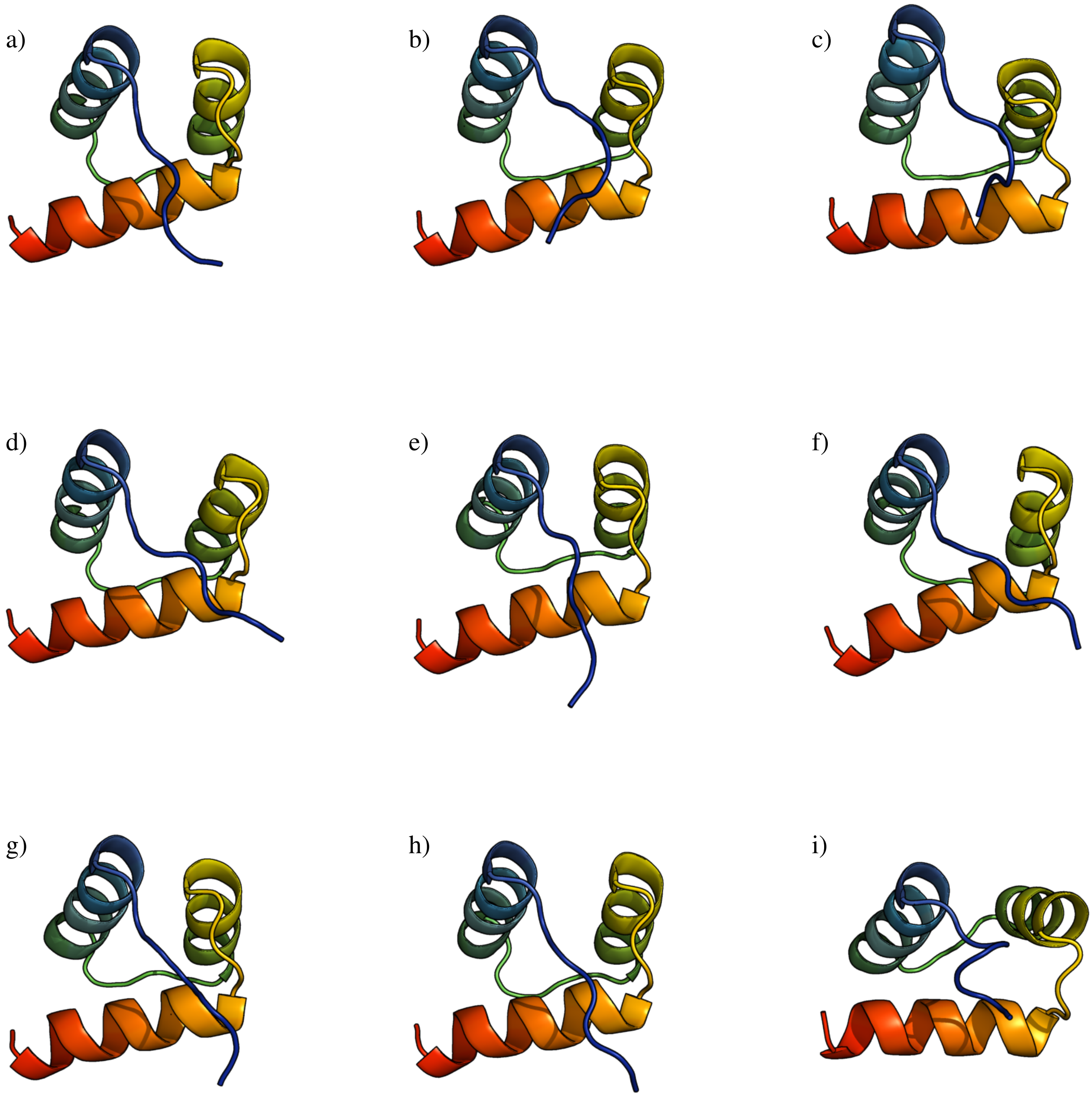

Figure S2. Lowest PROFASI+CamShift+HSEMM energy structures for ENHD. a) Crystal structure 1ENH. b) Cauchy / fixed. c) Cauchy / sampled. d) Gaussian / sampled. e) Gaussian / fixed. f) Gaussian / marginalized. g) Square well / $\alpha = 1$. h) Square well / $\alpha = 5$. i) CS-Torus

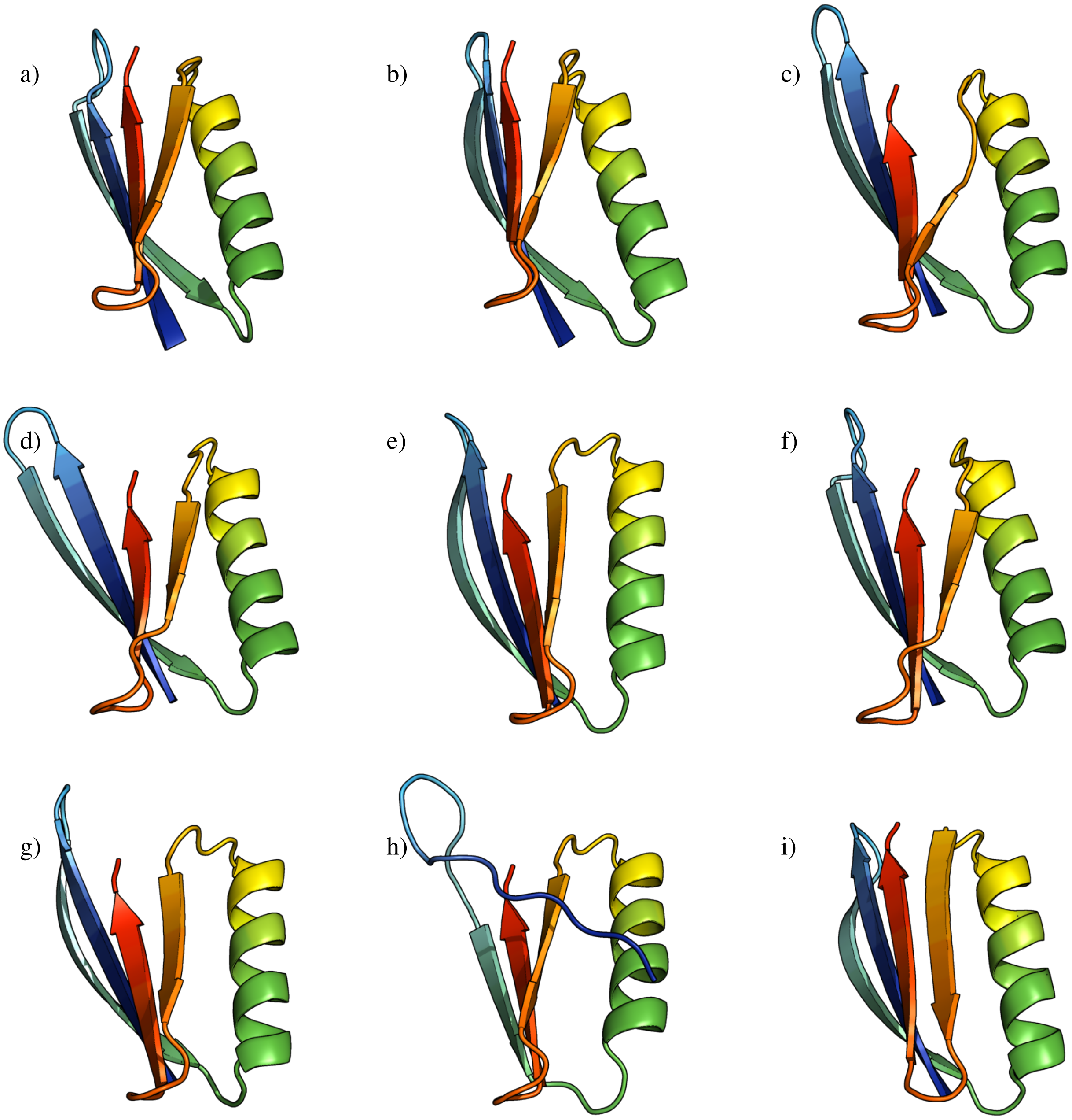

Figure S3. Lowest PROFASI+CamShift energy structures for Protein G. a) Crystal structure 2OED. b) Cauchy / fixed. c) Cauchy / sampled. d) Gaussian / sampled. e) Gaussian / fixed. f) Gaussian / marginalized. g) Square well / $\alpha = 1$. h) Square well / $\alpha = 5$. i) CS-Torus

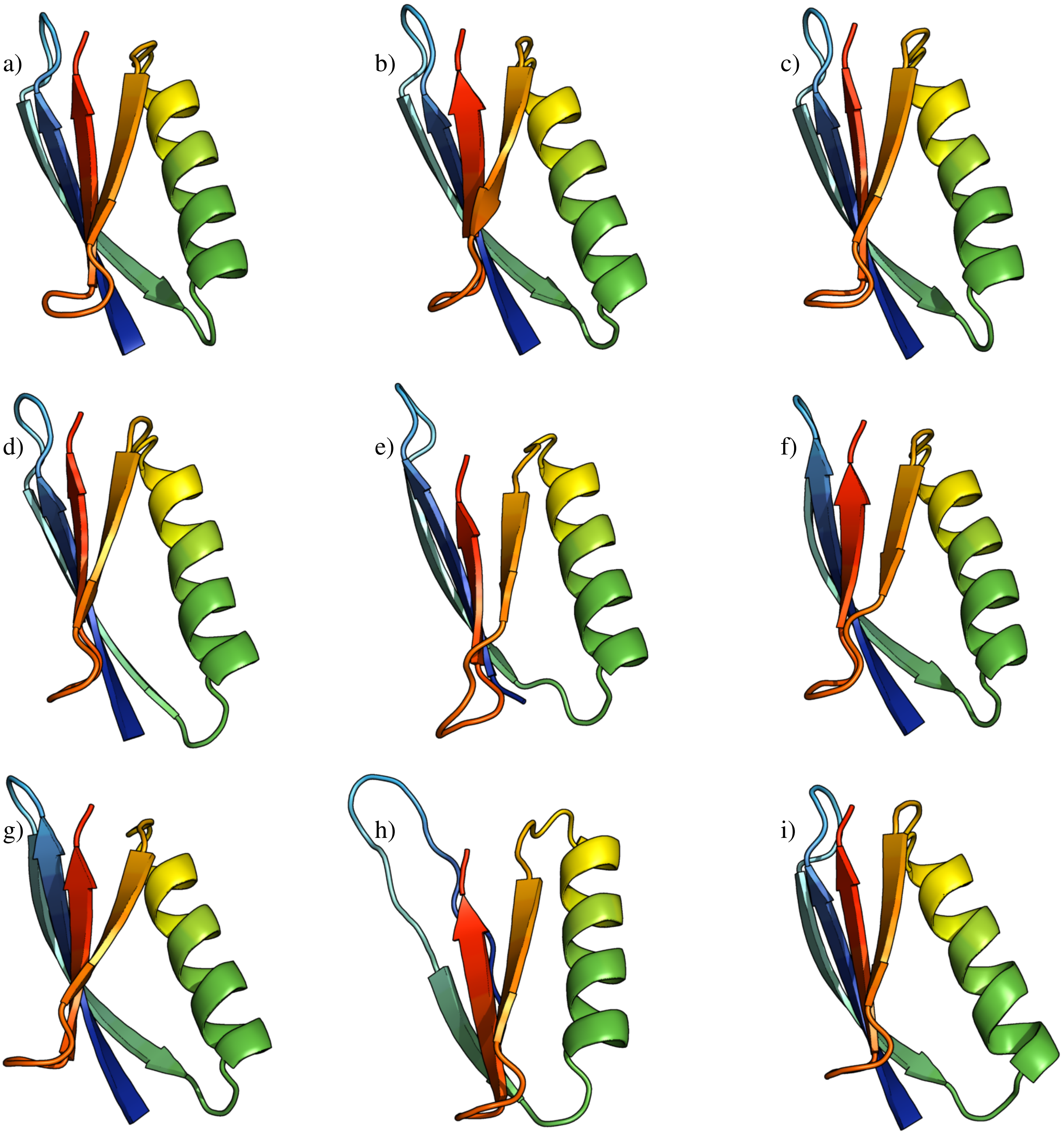

Figure S4. Lowest PROFASI+CamShift+HSEMM energy structures for Protein G. b) Cauchy / fixed. c) Cauchy / sampled. d) Gaussian / sampled. e) Gaussian / fixed. f) Gaussian / marginalized. g) Square well / $\alpha = 1$. h) Square well / $\alpha = 5$. i) CS-Torus

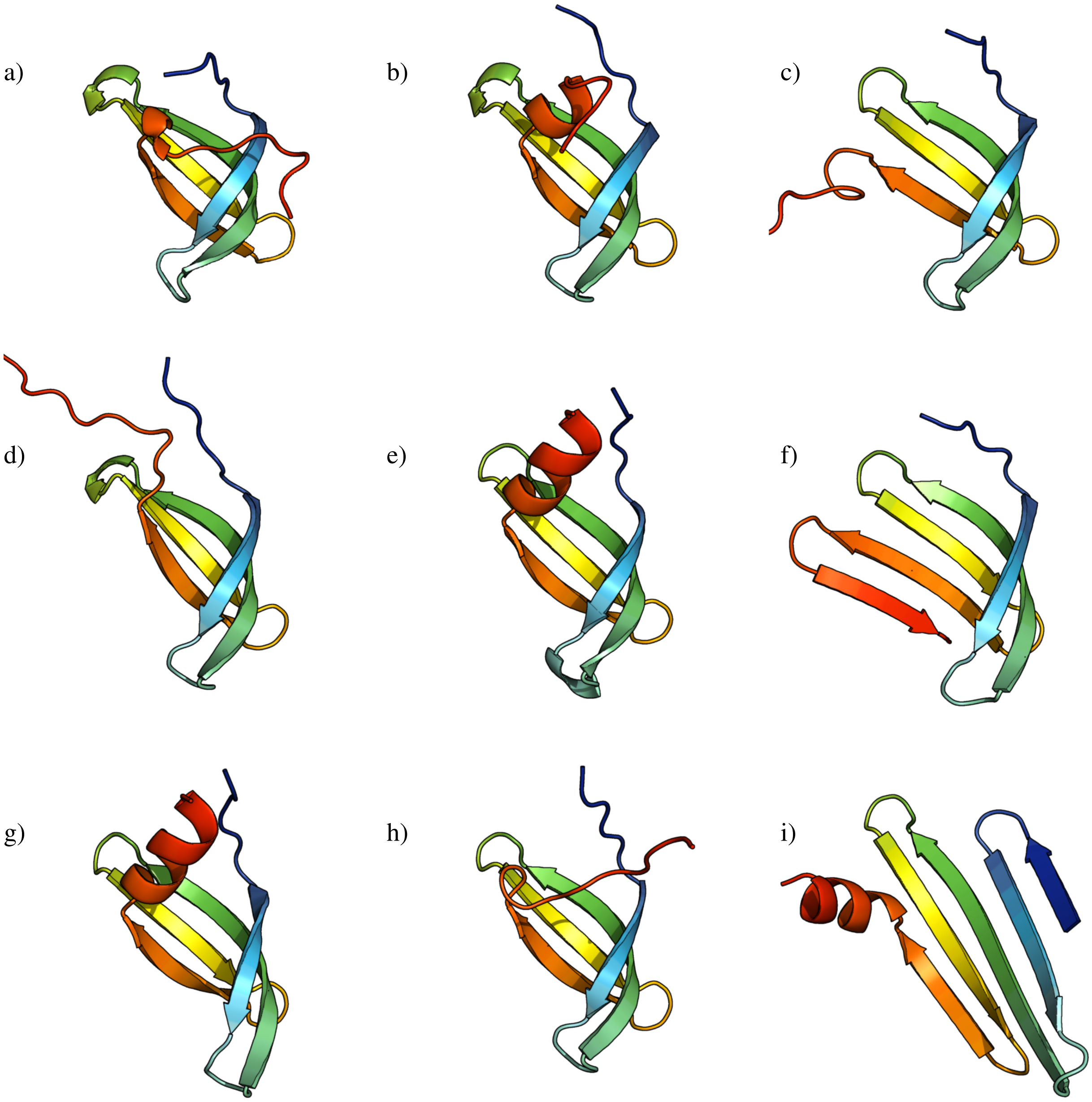

Figure S5. Lowest PROFASI+CamShift energy structures the SMN Tudor Domain. a) Crystal structure 1MHN. b) Cauchy / fixed. c) Cauchy / sampled. d) Gaussian / sampled. e) Gaussian / fixed. f) Gaussian / marginalized. g) Square well / $\alpha = 1$. h) Square well / $\alpha = 5$. i) CS-Torus

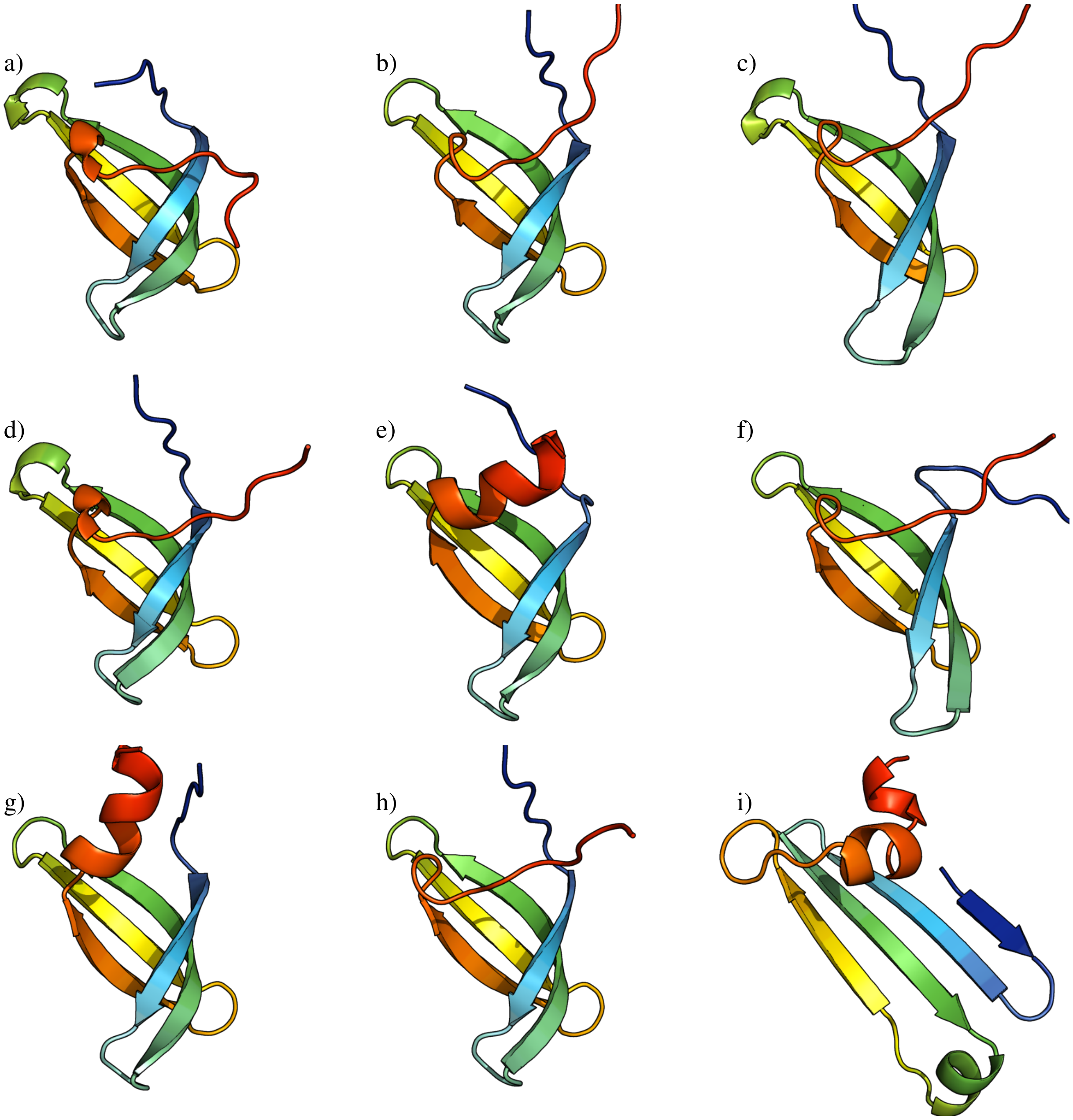

Figure S6. Lowest PROFASI+CamShift+HSEMM energy structures for the SMN Tudor Domain. a) Crystal structure 1MHN. b) Cauchy / fixed. c) Cauchy / sampled. d) Gaussian / sampled. e) Gaussian / fixed. f) Gaussian / marginalized. g) Square well / $\alpha = 1$. h) Square well / $\alpha = 5$. i) CS-Torus

PHAISTOS settings

Protein G convergence simulation with sampled weights for the Cauchy potential:

```
./phaistos --pdb-file protein_g.pdb
  --temperature 300 \
  --iterations 10000000 \
  --init-from-pdb 1 \
  --monte-carlo-metropolis-hastings 1 \
  --monte-carlo-metropolis-hastings-declash-on-reinitialize 0 \
  --move-none 1 \
  --move-none-weight 0.01 \
  --energy-camshift-cached 1 \
  --energy-camshift-cached-star-filename protein_g.str \
  --energy-camshift-cached-energy-type "cauchy" \
  --energy-camshift-cached-sample-weights 1 \
  --energy-profasi-cached 1 \
  --move-crisp-dbn-eh 1 \
  --move-crisp-dbn-eh-weight 0.25 \
  --move-sidechain-uniform 1 \
  --move-sidechain-uniform-weight 0.5 \
  --move-semilocal-dbn-eh 1 \
  --move-semilocal-dbn-eh-weight 0.25 \
  --backbone-dbn-torus-cs 1
```

Protein G structure determination simulation with fixed weights for the Gaussian potential:

```
./phaistos --pdb-file protein_g.pdb \
  --iterations 100000000 \
  --monte-carlo-muninn 1 \
  --monte-carlo-muninn-min-beta 0.6 \
  --monte-carlo-muninn-max-beta 1.1 \
  --monte-carlo-muninn-initial-max 50000 \
  --energy-camshift-cached 1 \
  --energy-camshift-cached-star-filename protein_g.str \
  --energy-camshift-cached-energy-type "gauss" \
  --energy-profasi-cached 1 \
  --move-crisp-dbn-eh 1 \
  --move-crisp-dbn-eh-weight 0.40 \
  --move-sidechain-uniform 1 \
  --move-sidechain-uniform-weight 0.5 \
  --move-backbone-dbn 1 \
  --move-backbone-dbn-weight 0.1 \
  --backbone-dbn-torus-cs 1
```

REFERENCES

Schrödinger, LLC (2010). The PyMOL molecular graphics system, version 1.3r1.
